# A Comprehensive Survey on Signcryption Security Mechanisms in Wireless Body Area Networks

**DOI:** 10.3390/s22031072

**Published:** 2022-01-29

**Authors:** Saddam Hussain, Syed Sajid Ullah, Mueen Uddin, Jawaid Iqbal, Chin-Ling Chen

**Affiliations:** 1 School of Digital Science, Universiti Brunei Darussalam, Jalan Tungku Link, Gadong BE1410, Brunei; saddamicup1993@gmail.com (S.H.); mueenmalik9516@gmail.com (M.U.); 2 Department of Information and Communication Technology, University of Agder (UiA), N-4898 Grimstad, Norway; 3 Department of Computer Science, Capital University of Science and Technology, Islamabad 44000, Pakistan; jawaid5825@gmail.com; 4 School of Information Engineering, Changchun Sci-Tech University, Changchun 130600, China; 5 Department of Computer Science and Information Engineering, Chaoyang University of Technology, Taichung 41349, Taiwan; 6 School of Computer and Information Engineering, Xiamen University of Technology, Xiamen 361024, China

**Keywords:** wireless body area networks, signcryption, healthcare

## Abstract

WBANs (Wireless Body Area Networks) are frequently depicted as a paradigm shift in healthcare from traditional to modern E-Healthcare. The vitals of the patient signs by the sensors are highly sensitive, secret, and vulnerable to numerous adversarial attacks. Since WBANs is a real-world application of the healthcare system, it’s vital to ensure that the data acquired by the WBANs sensors is secure and not accessible to unauthorized parties or security hazards. As a result, effective signcryption security solutions are required for the WBANs’ success and widespread use. Over the last two decades, researchers have proposed a slew of signcryption security solutions to achieve this goal. The lack of a clear and unified study in terms of signcryption solutions can offer a bird’s eye view of WBANs. Based on the most recent signcryption papers, we analyzed WBAN’s communication architecture, security requirements, and the primary problems in WBANs to meet the aforementioned objectives. This survey also includes the most up to date signcryption security techniques in WBANs environments. By identifying and comparing all available signcryption techniques in the WBANs sector, the study will aid the academic community in understanding security problems and causes. The goal of this survey is to provide a comparative review of the existing signcryption security solutions and to analyze the previously indicated solution given for WBANs. A multi-criteria decision-making approach is used for a comparative examination of the existing signcryption solutions. Furthermore, the survey also highlights some of the public research issues that researchers must face to develop the security features of WBANs.

## 1. Introduction

Wireless communications, distributed processing, micro-electro-mechanical systems, wireless sensor applications, and embedded systems have all contributed to a significant revolution in Wireless Sensor Networks (WSNs). A WSN is a collection of distributed sensors that monitor and record the physical conditions of the environment, then organize and transmit the data to a base station [1]. WSNs have been employed in a variety of applications, including medical surveillance and monitoring. Medical monitoring has grown in importance as a control system that provides real-time data and communication. The term “WBANs” refers to the use of WSN in medical applications. A WBAN is a special type of sensor network that uses the internet to connect patients with medical service providers to communicate vital health information [2]. WBANs is a critical wearable and implant network for health diagnostics, monitoring, and regulating actuators by sensing various important data from various wireless sensors (deployed in/over the body). It has several advantages, including location-independent monitoring, no effect on patients’ mobility, early disease identification and prevention, and remote patient help, among others. As a result, it’s ideal for continuous monitoring, providing for precise diagnosis and real-time feedback to medical experts [3].

WBANs are an Internet of Things (IoT) application that aims to improve the quality of patient services [4]. The IoT market is estimated to exceed 19 trillion USD in the next several years [5]. By 2025, it is estimated that about 100 billion IoT devices will be in use around the world, with an estimated economic worth of more than USD 11 trillion [6]. WBANs are one of the most capable wireless sensor technologies for health care, allowing users of healthcare systems to communicate real-time data for essential applications such as remote health monitoring, sports, home/patient care, emergency response, and early intrusion detection [7,8,9,10]. However, the lack of adequate data sharing protection in such a networking paradigm allows rogue users to execute illicit acts on sensitive medical data. As a result, there is a significant loss of sensitive data and user privacy, which has a significant impact on patients. For example, if a patient has a heart attack, a wearable gadget, or wireless sensors (deployed over or in his/her body) can detect it. Thus, in a public network, user and data protection is essential, allowing a doctor to begin therapy immediately [11,12].

An effective security framework is necessary to secure the security of the WBANs system. Authentication and confidentiality are two of the most important security concerns in WBANs that must be addressed. Security and authentication are generally addressed through the use of encryption and digital signatures [13]. It is common to use the sign-then-encrypt method when both encryption and signing are required at the same time. Complicated cryptographic procedures, on the other hand, are not possible due to the strict limitations associated with low-end WBANs sensing devices, including limited onboard energy and CPU capabilities. Due to the use of an amalgamated approach known as ‘signcryption’, it is possible to overcome such a stumbling block [14]. Moreover, because of its lower cost, it is far more appropriate for resource-constrained scenarios such as WBANs than the alternative of using signatures followed by encryption.

### 1.1. Communication Architecture of WBANs

Data communication could be separated into multiple layers of communication when considering the entire WBANs ecosystem. It should be emphasized that when a person in this scenario moves, his or her body may also be moving. As a result, the placement of the sensors involved in this scenario may fluctuate, implying that WBANs are not considered static. In general, the WBANs standard [15,16,17] recognizes three levels of communication:

#### 1.1.1. Tier-1: Intra-BAN Communications

The communication at this tier can be wired/wireless. Zimmerman [18] suggests this method of communication. Only the sensors and the sink are connected in intra-BAN communication [19]. This tier’s communication range is about 2 m in and around the human body. As the sensors are essentially positioned within this connection range, this tier is vital. This is why the manner of communication is limited in range. In this layer, ZigBee [20] and Bluetooth [21] are employed as communication technologies. Sensors monitor physiological attributes and send the data to a sink, which is positioned within this tier’s borders. The sink’s function is to process and transfer the data to Tier 2 [17,22,23].

#### 1.1.2. Tier-2: Inter-BAN Communications

In this layer, communication occurs between the sink and one or more Access Points. In another possibility, there could be infrastructure that installs Access Points, or the Access Points could be purposefully placed in a dynamic environment to properly manage emergency occurrences. The purpose of this tier is to provide interconnection between various forms of easily available networks, such as cell phone networks (or the Internet) and WBANs. This tier can leverage wireless technologies such as 3G/4G, cellular, ZigBee, Wireless Local Area Networks (WLANs), and Bluetooth [17,22,23].

#### 1.1.3. Tier-3: Beyond-BASN Communications

Metropolitan Area Networks (MANs) were the inspiration for this layer. The medical sensor is linked to the Internet or any other network that transports data to the recipients, allowing medical and health professionals to view the data. The individual who receives the gift could be a doctor or a nurse [23]. The information could potentially be saved in the patient’s database. As a result, the database plays a crucial role in Tier-3. The patient’s/profile, users as well as his/her medical history, is maintained in the database. When this happens, the doctor will receive a notice indicating that the patient’s condition is deteriorating, and the needed action can be taken using the database record before the patient arrives at the hospital [17,22].

The most essential components of Tier-3’s are the medical environment and database, which contain the user’s medical history and profile. As a result, doctors/patients can be notified of a medical emergency via the Internet or text messaging. Tier-3 additionally ensures that any important patient data that can be used for therapy is restored [23]. Depending on the application, the sink-in in Tier-1 can communicate with an AP through 3G/4G/GPRS instead.

**Figure 1:**
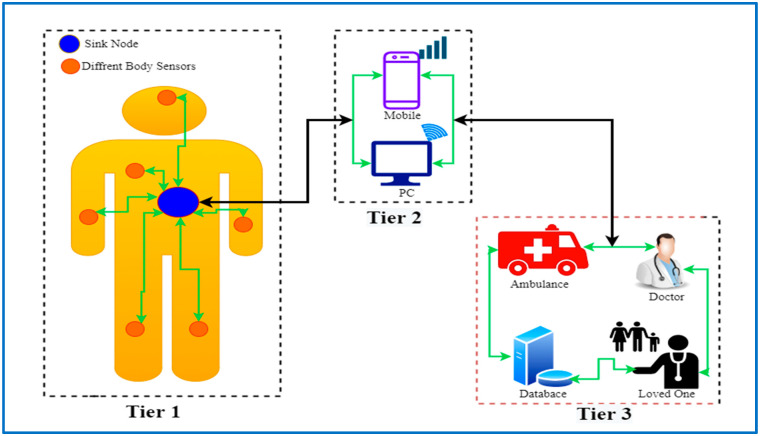
Communication Layers in the WBANs.

The necessary WBANs communication layers are depicted in Figure 1. In Tier-1 communication, two BANs are illustrated in the illustration, with on-body nodes and implanted nodes spread throughout the body. All nodes are either directly connected to the hub or through a relay node.

### 1.2. WBANs Applications

WBANs are being used in a variety of fields, including medical, entertainment, military, and sports [23]. WBANs have an important role in the medical industry, both in terms of saving lives and transferring patient information in an emergency [24]. WBANs entail the implantation of sensors on the human body that will monitor the patient’s health state in real-time. Any abnormal changes in the patient’s health, such as high fever, a low heart rate, or other symptoms, will be communicated to the doctor via the internet for prompt action [25]. An implantable sensor and a wearable sensor are the two types of WBANs applications that have been classified [26]. A sensor that is implanted into the human body with the use of surgery and is not meant to be removed from the patient’s body is known as an implantable sensor. When patients need to be monitored, a wearable sensor is used, which is worn by the patient and provides the necessary information. The Wearable Sensor node, on the other hand, assists in the identification of patient movement and abnormal positions. It is possible to remove wearable sensors from a patient’s body at any time. As an example, a wearable personal digital assistant can assist in the monitoring of blood glucose, body temperature, SpO2, the functioning of the heart, and blood pressure [27].

**Figure 2:**
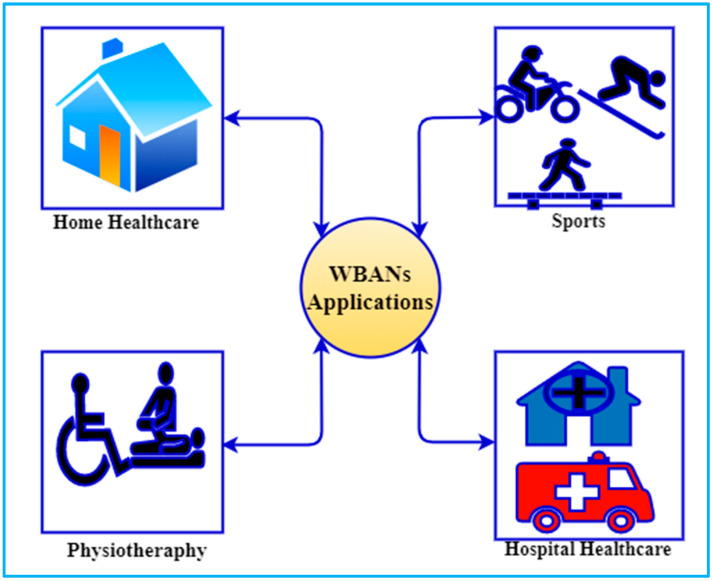
Application of WBANs.

WBANs are being used to develop a wide range of applications, including remote healthcare, ambient assisted living, and even user-centric applications like gaming and smart homes, as illustrated in Figure 2. In recent years, there has been a great deal of interest in the field of human activity recognition [28]. But there is a rapid expansion of the use of WBANs in healthcare applications, where, among other things, remote medical supervision could be advantageous for eldercare, early detection, and treatment of conditions including chronic diseases. The elderly might feel more independent in their daily routines with the support of ambient assisted living applications. Similarly, WBANs is useful in the entertainment industry because it aids in the transfer of data streaming operations.

In addition, WBANs are used to monitor a player’s practice as well as his or her physical fitness in sports such as hammer throwing, swimming, water volleyball, cricket, football, and other similar activities. By analyzing sensing data, it is possible to develop specialized measures to improve their performance while also maintaining their health [29]. Wearable sensors respond to body movement during water sports like swimming and water volleyball by switching communication media from air to water or vice versa. For such applications, a water-resistant sensor enclosure is required, as well as clever MAC protocols that can switch communication media on demand. In addition, WBANs are extremely important in the military since they allow medical personnel to monitor a soldier’s health and locate him in the event of an emergency.

Disaster relief and emergency response scenarios such as fire and flood rescue are expected to utilize WBANs in the future [30]. When body sensors are used in disaster relief, distress signals are sent that can be picked up by rescue equipment or relayed or supplied by neighbouring BANs [31]. As a result, WBANs applications now have an important new dimension that requires not only intra-BAN but also inter-BAN communication capabilities in a cross-medium environment. Different types of sensors, such as temperature sensors, multimedia sensors, and so on, are used in conjunction with GPS in these applications. As a result, the data size varies depending on the type of sensor used. The fact that flood rescue sensors can transmit data across water and air necessitates the adoption of smart MAC protocols.

Sensors are used in, on, or around the human body in all of these applications, and they also collect information about the user’s behaviour. Therefore, humans are inextricably linked to the system, raising concerns about its overall security and reliability. For example, data integrity is a critical requirement for WBANs applications because incorrect information about a person’s body vitals could result in incorrect treatment and, as a result, fatal consequences. It is also critical for these applications to protect user data confidentiality because sensitive information about user behaviour and their daily lives could be revealed, which could pose a threat to their social well-being. Even the slightest bit of information or misinformation about a player’s fitness has the potential to harm their reputation. Consequently, WBANs applications should be made more secure overtime to assure the precision and long-term durability of the monitoring applications for which they are designed. It is becoming increasingly vital to set rigorous security requirements as more and more parties become involved with such applications.

### 1.3. Authors Motivation and Contributions

Recently, there has been a lot of interest in authentication research in the WBAN’s security field. To improve the security of WBANs, plenty of comprehensive survey and analysis of the existing state-of-the-art authentication approaches has been proposed in the literature. However, authentication, as well as confidentiality, are important aspects of WBANs security. Unfortunately, none of the existing surveys cover signcryption (authentication, confidentiality) solutions. Table 1 presents a summary of qualitative comparisons between previous surveys and the proposed survey. Following are some of the major contributions.

- A quick overview of WBANs technology, applications security requirements, and architecture that provides readers with a basic understanding of the research domain.
- To the best of our knowledge, the current study surveys all signcryption approaches proposed for securing WBANs infrastructure. Additionally, the schemes have been thoroughly examined, analyzed, and compared.
- Based on the methods utilized, this survey classifies existing signcryption schemes into six categories: Attribute-based signcryption schemes, Identity-based signcryption schemes, PKI-based signcryption schemes, Certificateless signcryption schemes, Certificate-based signcryption schemes Heterogeneous signcryption schemes. Additionally, each scheme’s strengths and flaws are assessed and compared to the others.
- This survey not only gives a thorough examination of the existing signcryption schemes for WBANs security and privacy criteria but also detailed explanations of the attacks that target these schemes.
- Qualitative analysis of related surveys is carried out to show the novelty of the proposed survey.
- Future research directions, opportunities, and open issues have been offered.

### 1.4. Paper Organization

**Figure 3:**
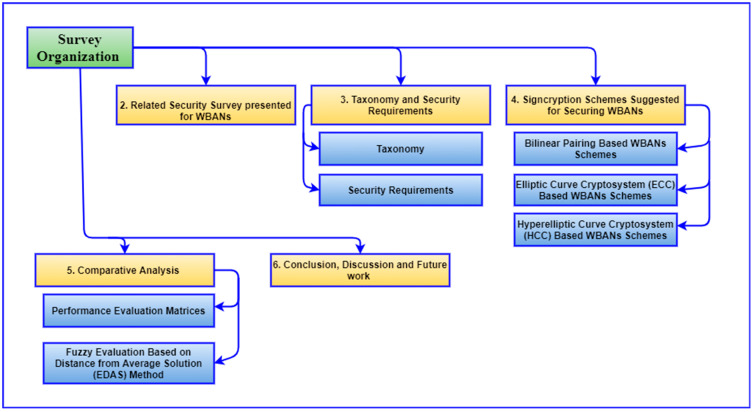
Survey Organization.

The rest of this survey is divided into seven sections, which are listed below. Section 2 provides the summary of WBANs security surveys. In Section 3, security requirements and taxonomy of WBANs signcryption schemes based on the type of cryptography were discussed. In Section 4, the efficiency of the signcryption schemes is compared based on computation time, communication overhead, security hardness, and security strength. Section 5 outlines WBAN’s future research possibilities and directions with a conclusion as shown in Figure 3.

## 2. Related Security Survey Presented for WBANs

**Figure 4:**
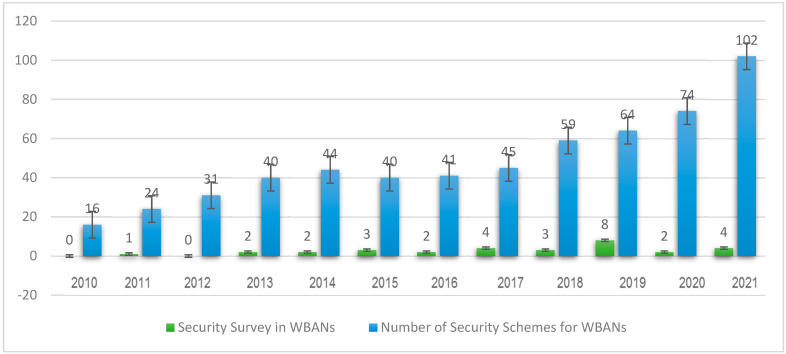
WBANs Security Survey and Schemes from 2010 to 2021.

**Figure 5:**
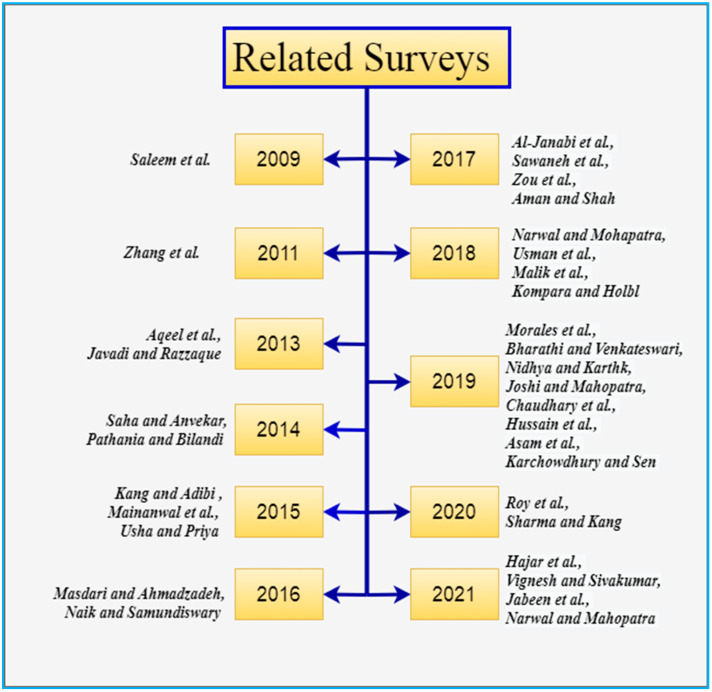
Related Security Surveys in the Domain of WBANs.

The primary goal of this review study is to provide an overview of the most recent signcryption security research papers as well as upcoming trends in WBANs security. Through Figure 4, the authors’ process for selecting appropriate research papers relevant to the survey is depicted in diagrammatic form. The research keywords that were used in the search selection: “WBANs security, WBANs security survey, WBANs security requirements, and WBANs application”. The relevant information about our research is dispersed across the various conferences, chapters, and journals that have been published in the past. To extract relevant materials, the most widely used online repositories, such as IEEE Explore, Springer, Science Direct, etc. A manual search in the relevant area was also carried out as a second step. Besides, we reviewed all the security surveys (to the best of our knowledge) in the domain of WBANs as shown in Figure 5.

In 2009, Saleem et al. [32] highlighted the main security requirements and Denial of Service (DDoS) concerns in WBANs. In addition, the authors provide a broad overview of security essentials and highlight existing WBANs threats at several layers. Finally, the authors give a thorough assessment of existing security protocols for WBANs.

In 2011, Zhang et al. [33] attempted to investigate the probable resource-constrained WBANs attacks and present a review of communication protocols, cryptographic algorithms, and key management procedures pertinent to the security of WBANs. The authors also examine existing solutions’ flaws and probable future research areas in WBANs security.

In 2013, Aqeel et al. [34] attempted to offer a critical analysis of potential WBANs authentication techniques. The IEEE 802.15.6 standard is used to guide the discussion and reviews. In WBANs, Javadi, and Razzaque [35] examine major security and privacy issues as well as potential threats. The authors also discuss an unsolved Quality of Service (QoS) problem in WBANs that has the potential to cause major security difficulties. Finally, the authors outline future directions that could be pursued.

In 2014, Saha and Anvekar [36] presented a state-of-the-art in existing WBANs security aspects. Additionally, the authors also highlight several significant security challenges. Pathania and Bilandi [37] give an outline of WBANs and related challenges, with a focus on the security issue. The authors also discuss security attacks in WBANs and security necessities in WBANs, as well as a vulnerability assessment.

In 2015, Kang and Adibi [38] investigated the security features of application and communication protocols. The authors also discuss the architecture, vulnerabilities, and attacks, as well as future opportunities. Mainanwal et al. [39] summarized the benefits and drawbacks of different security and privacy solutions used in WBANs. The threats and constraints that WBANs face is also discussed. Finally, a discussion on possible future research directions is held. Usha and Priya [40] address various types of attacks, prevention strategies, and simulation tools for WBANs.

In 2016, Masdari and Ahmadzadeh [41] conducted a comprehensive review and analysis of the numerous authentication schemes offered in the literature to increase the security of WBANs. Furthermore, the authors discuss the benefits and drawbacks of various authentication techniques, as well as a full comparison of their features and capabilities. Finally, the authors outline future directions that could be pursued. A broad overview of WBANs and WSNs is presented by Naik and Samundiswary [42]. In addition, the authors discuss WBAN security protocols, including their advantages and disadvantages.

In 2017, based on recent publications and standards, Al-Janabi et al. [43] examined the communication architecture of WBANs, as well as the security and privacy needs, security threats, and the major issues that these systems face. The survey also includes information on the most up-to-date security measures and studies in WBANs. Finally, potential topics for future research and development are investigated. A survey report by Sawaneh et al. [44] focuses on building and implementing WBANs in healthcare systems. In addition, the authors provide a brief overview of WBAN security and privacy requirements. Zou et al. [45] examine the applicability of a variety of secure communication technologies within WBANs and between external organizations and WBANs. Furthermore, their research emphasizes the importance of primary security requirements for secure transmission at both levels. Aman and Shah [46] conduct a thorough review of significant studies on mobile, ubiquitous, and WBANs, focusing on routing and security challenges.

In 2018, Narwal and Mohapatra [47] attempted to provide a comprehensive analysis of several authentication approaches. The authors also provide a complete analysis of the schemes based on security attacks, security features, and a variety of other factors. Usman et al. [48] provide a succinct overview of WBAN security. The authors suggest a taxonomy that provides a simple manner of classifying entities involved in healthcare systems. Security issues have been investigated at all WBANs layers. The authors have done an excellent job of identifying outstanding topics and potential research directions. Malik et al. [49] present a broad overview of major security requirements and potential attacks in WBANs at various layers of the OSI model. After providing an overview of WBANs for healthcare monitoring, the survey addresses cryptographic solutions for addressing security and privacy issues. Kompara and Holbl [50] focus on the security and key agreement of intra-BAN communication. It gives a thorough analysis of existing key agreement methods and categorizes them into four groups: classic, physiological value-based, secret key-based, and hybrid key-based schemes. In addition, each class is described, and the security of WBANs against threats is assessed.

In 2019, Morales et al. [51] proposed several WBANs design solutions as well as a detailed assessment of security services. Overall, the survey aims to provide a holistic security picture of the entire WBANs system. Bharathi and Venkateswari [52] give a general overview of WBANs, their applications, and security concerns. Based on the most recent evaluations and publications, many security issues, and responses in WBANs are discussed. A systematic literature evaluation on the security and privacy issues of electronic healthcare record systems in WBANs is presented by Nidhya and Karthk [53]. WBANs Authentication protocols have design issues, according to Joshi and Mahopatra [54]. In addition, the authors suggest important prospects for research communities. Chaudhary et al. [55] explore the security and privacy difficulties with WBANs, provide remedies, and describe the type of authentication technique employed. Hussain et al. [56] provide an overview of WBANs and their properties, as well as numerous authentication types and schemes classification. It also compares and contrasts various authentication techniques, highlighting their advantages, disadvantages, performance evaluation, and robustness against various security attacks. Finally, the authors outline future directions that could be pursued. Asam et al. [57] present a thorough assessment of the issues in WBANs from the perspectives of communication and security. Regrettably, the authors provide only a cursory review while ignoring major security concerns. In a WBANs study, Karchowdhury and Sen [58] look at major security requirements and Denial of Service concerns.

In 2020, Roy et al. [59] presented a comprehensive analysis of WSNs and WBAN’s security and privacy challenges. The authors examine the characteristics, architecture, performance measures, and applications of both in-depth, and then conduct a comparative analysis. Finally, researchers are offered open research challenges. Sharma and Kang [60] examine and evaluate WBAN’s routing, security, energy, and cost-cutting problems.

In 2021, Hajar et al. [61] give a complete overview of WBANs technology with a special focus on security and privacy concerns and countermeasures, as well as proposed research directions and open issues. The authors, on the other hand, were only interested in authenticating schemes. Vignesh and Sivakumar [62] cover numerous security procedures and routing issues that WBANs face, as well as attacks that could occur through the network and a review of some of the mechanisms that are in place to prevent them. The authors also look into the security of various attack scenarios. Finally, the study summarizes the primary challenges the users encounter while creating a network in WBANs, which is a new branch of science in the face of the pandemic. A systematic literature review of the different security approaches for WBANs is presented by Jabeen et al. [63]. The authors identify research topics to investigate the feasibility of multiple attacks while keeping memory restrictions in mind. To guarantee that the schemes are relevant to the research subject, a quality assessment is undertaken. Furthermore, the schemes are considering from 2016 to 2020 to focus on recent work. Several existing techniques are investigated in the literature to see how the security of transmitting patients’ healthcare data might be improved. Based on relevant qualities, data security techniques using AES, ECC, SHA-1, and hybrid encryption are evaluated. Finally, the authors assess security in the context of several attack scenarios. Narwal and Mahopatra [64] outline and discuss various security and authentication schemes and solutions. Unlike earlier surveys that have looked at security and authentication in WBANs in a piecemeal fashion to cover main research topics, this study has taken a holistic approach to security and authentication in WBANs. A detailed assessment of security essentials, security risks, attackers and their attack techniques, and presently available countermeasures have been provided, as well as a complete description of security mechanisms in WBANs. The authors also examine the uses of WBANs, open research challenges, recommendations, and future developments. Overall, the study delves into WBANs functionality, technology, building blocks, and a much broader picture of WBAN’s security and authentication.

WBANs are a well-established research topic that has been around for a while. As a result, numerous overview and survey papers have been published in the field, compiling research on various aspects of the field. The surveys mentioned above are primarily aimed at authentication, architecture, security, and challenges, among other things. Security requirements, applications, signcryption schemes, the classification of existing signcryption schemes based on the type of cryptography and algorithm, an overview of newly introduced schemes, a compiled list of schemes’ security properties, and an overview of methods for security and performance evaluations are all included in this paper’s contributions.

The fundamental purpose of this study is to create a clear and thorough classification, analysis, and comparison of the WBAN signcryption schemes. As compared to the previously mentioned studies, this survey includes (i) an in-depth analysis of how well each signcryption scheme fulfills the security requirements of a WBANs; (ii) detailed information about which specific security requirements are addressed by signcryption schemes; and (iii) an in-depth analysis of how well each signcryption scheme performs in terms of computational time, communicational overheads, and security strength. Table 2 summarizes the qualitative comparison of previous surveys with the proposed.

## 3. Taxonomy and Security Requirements

Here in this section, we will discuss the taxonomy of the signcryption schemes of WBANs based on the type of cryptography used as well as the security requirements of WBANs signcryption schemes.

### 3.1. Taxonomy

**Figure 6:**
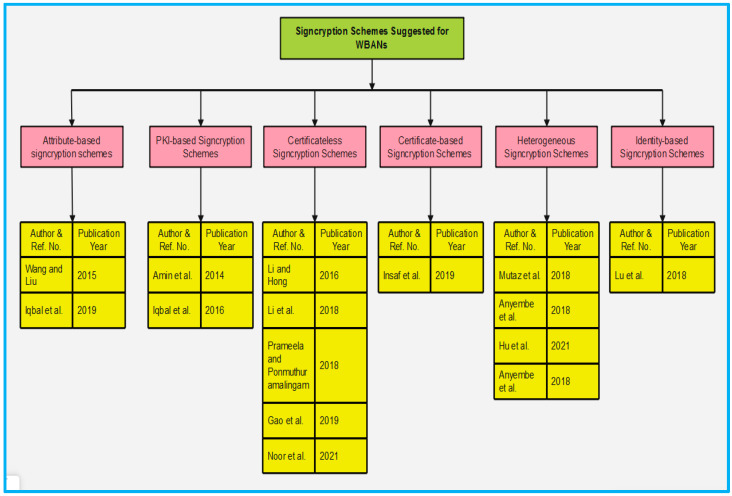
Taxonomy of WBANs Signcryption Schemes.

Signcryption is one of the most important aspects of security for establishing trust between humans and medical experts. The implementation of correct signcryption schemes ensures a WBAN’s security while also making it easier to identify non-legitimate users and false messages. To overcome problems and provide secure communication in WBANs, many researchers have suggested signcryption schemes. The majority of signcryption schemes rely on various cryptographic techniques. Attribute-based signcryption [65] schemes, PKI-based signcryption schemes, Certificateless signcryption [66] schemes, Certificate-based signcryption schemes, Identity-based signcryption schemes, and Heterogeneous signcryption schemes are the five types of schemes classified in this survey. The following methods are linked by the fact that they all use cryptography, as seen in Figure 6. The existing schemes have been evaluated in terms of their ability to meet security and performance requirements (computation time and communication overheads). The performance parameters tabulated in Section 5.1 which were used in this survey to define the computation time and communication overheads are based on the work.

### 3.2. Security Requirements

**Figure 7:**
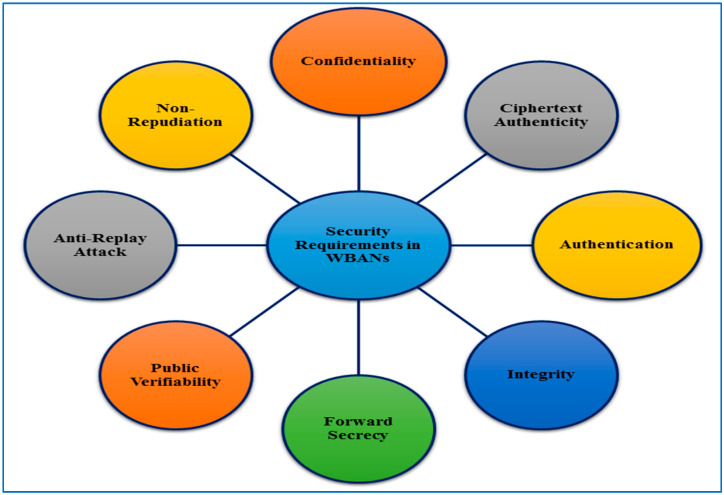
Security Requirements for WBANs.

To maintain the security of a patient’s health records at all times, the WBANs system necessitates the implementation of certain security measures. Specific security measures must be implemented in a supporting WBANs architecture to ensure all of these aspects. Within each WBANs system, the security of patient information is very critical. When data is sent, collected, processed, and safely kept, it must be protected from unauthorized users. Figure 7 depicts some of the critical security criteria for WBANs. The following are the primary security considerations for ensuring the safety of a WBANs system and its widespread acceptance by its users.

Confidentiality, authentication, integrity, and non-repudiation are at least four security qualities that should be met by communication between the user and the controller. Except for the user and the controller, confidentiality keeps query messages secret. Only the authorized user has access to the WBANs, thanks to authentication. Integrity ensures that a user’s query message has not been tampered with by unauthorized parties. Non-repudiation prevents the user’s past inquiries from being denied. That is, the WBANs cannot deny the user’s action if the user has sent a query message to it. We also hope that this communication meets the requirements for public verifiability and ciphertext authenticity. A third party can check the authenticity of ciphertext without knowing the controller’s private key, which is known as public verifiability. The term “ciphertext authenticity” refers to the ability of a third party to check the correctness of ciphertext without having to decrypt it. An attacker cannot replay existing messages if the sender and receiver use fresh nonce and time stamp techniques commonly termed as an anti-replay attack. Forward secrecy is a term used to describe the practice of keeping information hidden from Even if the intruder has the access to the private key of the sender’s, he or she will not be able to obtain the encryption/decryption keys. Forward secrecy occurs when an attacker is unable to access the user’s encryption/decryption key.

## 4. Signcryption Schemes Suggested for Securing WBANs

**Figure 8:**
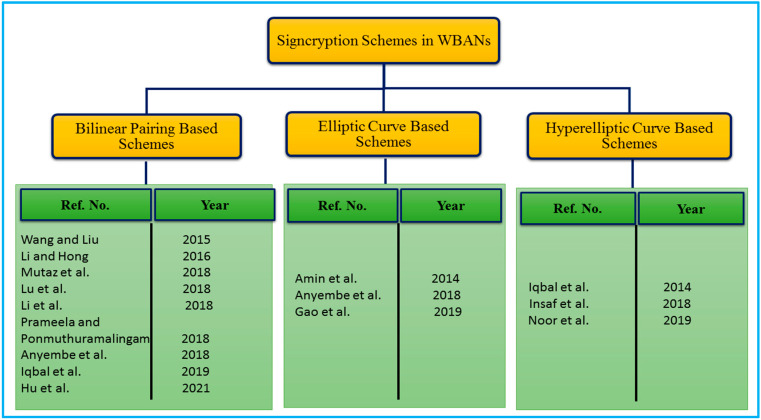
Hardness Algorithm Based Taxonomy of the WBANs Signcryption Scheme.

We investigated existing WBANs encryption strategies in terms of hardness algorithm, security features, computing time, and communication overhead in this part. Table 3, Table 4 and Table 5 summarize the contributions, advantages, and disadvantages of existing signcryption techniques for WBANs. The following is a debate that follows a critical assessment of existing schemes. Furthermore, Figure 8 shows the hardness algorithm-based taxonomy of the WBANs signcryption schemes.

### 4.1. Bilinear Pairing Based WBANs Schemes

Let $G_{1}$ and $G_{2}$ denote a cyclic additive and cyclic multiplicative group. The prime order q is used in all of these groups. The points $P\in \;G_{1}$ computes the $G_{1}$. Consider $e:G_{1}*G_{1}\to G_{2}$ as a bilinear pairing that satisfies the following key features [67,68].

#### 4.1.1. Bilinearity

For all $P,\;S,\;R\in G_{1},\;e\left(P+S,R\right)=e\left(P,R\right)\;e\left(S,R\right)\;\&\;\left(P,\;S+R\right)=e\left(P,S\right)\;e\left(P,\;R\right).$ Likewise, with all $a,\;b\;\in Z_{q}^{*},\;e\left(aP,\;bP\right)=e\left(P,P\right)ab=e\left(P,\;abP\right)=e\left(abP,P\right)$.

#### 4.1.2. Non-Degeneracy

Given two points $P,\;S\in G_{1}$ such that e(P, S)≠1 or e(S, R)≠e(P, P), where 1 denotes the $G_{2}$ group’s identification item

#### 4.1.3. Computability

A robustness approach for calculating (P,S) with all $P,\;S\in G_{1}$ should be available.

In 2015, using an attribute-based cryptosystem, Wang and Liu [69] proposed a ring signcryption approach for WBANs. The computational assumptions of bilinear pairing were responsible for the scheme’s security and efficiency. According to the authors, the design scheme satisfies a variety of security requirements, including authenticity, confidentiality, and non-repudiation, among others. However, the proposed scheme fails to address the issue of key escrow because the hospital authority serves as a private key generation center, generating private keys for data users and controllers. As a result, the hospital authority can easily forge the signature using the user’s private key, rendering the scheme ineffective. The efficiency of the scheme is also dependent on bilinear pairing, which may be jeopardized by higher computing power consumption and the increased nature of communication bandwidth, both of which are undesirable. The design scheme is also vulnerable to forward secrecy, mutual authentication, anti-replay attack, and public verifiability attacks, among others.

In the same year, Li and Hong [70], construct an access control and signcryption approach for WBANs using a certificateless cryptosystem. The computational assumptions of bilinear pairing were responsible for the scheme’s security and efficiency, and they were proven to be correct. According to the authors, the design scheme satisfies a wide range of security requirements, including authenticity, confidentiality, and non-repudiation, among others, and is therefore widely applicable. In contrast, because the hospital authority also serves as a private key generation center, generating private keys for data users and controllers, the proposed scheme does not deal with the issue of key escrow. Consequently, hospital authorities can easily forge the signature by using the user’s private key, rendering the scheme ineffective and rendering the scheme ineffective. Furthermore, the efficiency of the scheme is dependent on bilinear pairing, which may be jeopardized by increased computing power consumption as well as the increased nature of communication bandwidth, both of which are undesirable outcomes. In addition, the design scheme is vulnerable to attacks such as forward secrecy, mutual authentication, anti-replay attack, and public verifiability attack, among other types of vulnerabilities.

In 2018, Mutaz et al. [71] proposed a new IoT strategy based on heterogeneous signcryption, in which the sensor devices utilize certificateless infrastructure while the server utilizes public key infrastructure. Authentication, non-repudiation, integrity, and confidentiality are among the security properties claimed by the authors, and they demonstrate these properties using the ROM to prove the scheme’s security requirement. They also demonstrate how this technique can be applied in WBANs. This approach, however, may encounter difficulties with secret key distribution, certificate revocation, and administration as a result of the use of certificateless cryptography and public key infrastructure. Bilinear pairing is also used for security hardness, which results in increased consumption of computation resources as well as increased communicational overhead, which can be detrimental. In addition, there is a lack of mutual authentication, public verifiability, forward secrecy, and anti-replay attack mechanism in place.

In 2018, Lu et al. [72] suggested an attribute-based signcryption technique for a social network-based mobile healthcare system. To protect patients’ sensitive information, the authors use a four-party paradigm. For a range of studies, the authors claim that the offered approach achieves the security features of traceability, privacy, unforgeability, and accuracy. Moreover, the authors also claimed to have improved the efficiency by employing signcryption. However, because of the private key generator principle, this scheme may experience issues with private key distribution and key escrow. It is also open to forward secrecy, public verifiability, non-repudiation, mutual authentication, and anti-replay attack protection. Furthermore, bilinear pairing is used for security hardness, which can result in higher consumption of computational resources and greater communicational bandwidth.

Li et al. [73] present a unique technique based on certificateless signcryption, which they subsequently use to implement access control services in WBANs. Authenticity, integrity, confidentiality, non-repudiation, and anonymity were among the security aspects that the authors sought. The authors also compare their plan to other schemes and stats that they produce better outcomes in terms of energy use and computing costs. However, due to the Certificateless nature, this technique may have a partial private key distribution difficulty, as well as higher computing power consumption and a greater bandwidth nature due to the practice of bilinear pairing. It may also be affected as a result of a lack of public verifiability, forward secrecy, and mutual authentications.

For the aim of access control in WBANs, Prameela and Ponmuthuramalingam [74] suggested a better approach based on the concept of certificateless signcryption with anonymous mutual authentication and cost-efficiency. Secure authentication is achieved through the use of a Chaos baker map technique, which includes an XOR operation and a one-way hash chain function. According to the findings of the solution testing, the provided scheme beats earlier schemes in terms of, end-to-end delay, energy consumption, packet delivery ratio, throughput, and coverage time. Due to the certificateless cryptography notion, however, this technique may face partial private key distribution issues, as well as snootier computational power consumption and a higher bandwidth nature due to bilinear pairing. This technique can be harmed by a lack of forwarding secrecy, public verifiability, and anti-replay assault.

In 2018, Anyembe et al. [75] presented a heterogeneous signcryption-based keyword search technique for WBANs, in which the data owner employs certificateless cryptography while the server and receiver use public key infrastructure features. The given scheme was designed based on bilinear pairing mathematical structure. With this approach, the author claims security services such as secrecy, unforgeability, non-repudiation, and authenticity. Yet, due to bilinear pairing, the system may incur higher computational and communication costs, while it may also be hampered by the necessity for a safe route for the data owner distribution of partial keys and public key infrastructure certificate maintenance on the receiver and server sides. In addition, lack of forward secrecy, mutual authentication, and public verifiability can have an impact.

In 2019, Iqbal et al. [76] proposed a new BSN concept based on attribute-based cryptography and blockchain. The design scheme’s security and efficiency are based on bilinear pairing. Furthermore, the authors claim that the given scheme ensures security aspects such as confidentiality, unforgeability, anti-replay attack, and resistance to a man-in-the-middle attack by utilizing less energy, computational consumption, and communication overhead. However, due to bilinear pairing, the technique may incur higher computational and communication costs, while certificateless cryptography and public key infrastructure may require the usage of the secure channel for the distribution of partial keys and certificate administration, certificateless cryptography and public key infrastructure may not. Lack of security criteria such as mutual authentication, public verifiability, and forward secrecy can also be detrimental.

In 2021, Hu et al. [77] suggested a heterogeneous solution for WBAN that rely on an equality test to migrate from identity-based to public key infrastructure. Before it is uploaded to the cloud server, identifiable information is encrypted by the sensors in the identity-based cryptography system, which is protected by the public key of the management center in the public key infrastructure system. To make matters worse, the proposed scheme makes use of bilinear pairing to increase security hardness, which is a computationally intensive operation.

#### 4.1.4. Lesson Learned, Discussion, and Open Challenges

Based on our findings from the literature stated above and (Table 3), each technique has its own set of advantages and disadvantages, making it difficult to determine which technique is preferable to the others. Furthermore, each of them has its own set of security constraints based on security requirements including confidentiality, unforgeability, integrity, anonymity, non-repudiation, forward secrecy, public verifiability, and replay attack, among others. The WBANs nodes, as obvious, are limited in terms of power efficiency, memory, and computing and communication capabilities. Due to power consumption, cryptographic operations necessary for computations, communication, and storage must be severely limited. While Pairing-based techniques are considered to be costly cryptography primitive. As a result, we believe that schemes based without pairing would be more efficient in the long run. Secondly, all of the proposed techniques are based on pairing and have been demonstrated to be secure using ROM. Besides, in terms of security requirements, the scheme presented by Wang and Liu [69] has been subjected to forward secrecy, mutual authentication, public verifiability, and anti-replay attack. The Li and Hong [70] scheme suffer from forwarding secrecy and anti-replay attack flaws. The lack of forward secrecy, public verifiability, anti-replay attack and mutual authentication can all impair the Mutaz et al. [71] method. The Lu et al. [72] technique has the flaw of forwarding secrecy, non-repudiation, and anti-replay attack. The approach by Li et al. [73] does not provide forward secrecy, public verification, or mutual authentication. Prameela and Ponmuthuramalingam’s [74] method has been plagued by the lack of forwarding secrecy, anti-replay attack, and public verifiability assault. Anyembe et al. [75] describe a technique that lacks security features such as forward secrecy and mutual authentication. The technique used in [77] lacks both public verification and forward secrecy, which can be troublesome. Similarly, forward secrecy, public verifiability, anti-replay attack and mutual authentication are all missing from the Iqbal et al. [76] approach. However, proposing a novel strategy that is secure in the standard model using pairings remains an unresolved challenge. Additionally, the compact scheme that can achieve all the security requirements is still open.

### 4.2. Elliptic Curve Cryptosystem (ECC) Based WBANs Schemes

This section will quickly outline the principles of ECC and the resulting computational hardiness [78,79], which provides security and can survive a wide range of threats.

Let $F_{p}$ be the finite field with prime order p. A non-singular is defined by the ECC $y^{2}=x^{3}+ax+b\;mod\;p$, where $4a^{3}+27b^{2}\neq 0$ & $a,\;b\;\in F_{p}$. Consider O to be the infinite point. With order q and generator P, all of the points form an additive group G. 

#### 4.2.1. Elliptic Curve Discrete Logarithm Problem (ECDLP)

On ECC, there are two random points P and S such that (P, S) ∈ G, where P∈G calculates the G with a big primer order q. The ECDLP’s fundamental idea is to use S=xP∈G, to calculate an integer x, where $x\in Z_{q}^{*}$ is an unknown integer.

Based on symmetric cryptography incorporating Signcryption, For Body Area Networks, Amin et al. [80] propose a hybrid key management technique. By merging cluster head selection and session key generation into a single logical process, the authors claim to reduce computation time and communication overhead. According to the authors, the design scheme can achieve secrecy, Integrity, authentication, and anti-replay attack among other security aspects. Unfortunately, the authors utilize ECC, the approach may suffer from higher computing power consumption and increased bandwidth. It may also be affected by the issues with certificate renewal and revocation, as well as a lack of forward secrecy, public verifiability, anti-replay attack and mutual authentication.

In 2018, Anyembe et al. [81] suggested a Heterogeneous signcryption-based access control solution for WBANs, in which the controller uses the notion of certificateless cryptography and the identity-based idea is used by the application service providers. The cost and security hardness of the proposed scheme are determined by the mathematical foundation of the ECC. The authors of this technology state that the given scheme is more cost-effective, as well as provides security services such as forward secrecy, public verifiability, anti-replay attack and mutual authentication. Nevertheless, the use of ECC may result in higher computational and communication costs for the scheme, while the requirement for a secure path for the application provider’s partial private key distribution and the controller’s key escrow problem may make it difficult to complete the task in some cases. A lack of public verifiability, forward secrecy, and mutual authentication can all have an impact on the security of a scheme.

In 2019, using ECC for WBANs access control, Gao et al. [82] proposed a certificateless signcryption approach. According to the author, the given scheme provides the security services of secrecy, unforgeability, non-repudiation, and authenticity while also being cost-effective. However, due to the use of ECC, the technique may result in higher computational and communication costs, while the requirement for a secure route for partial private key distribution may have an impact on the technique. A lack of forwarding secrecy, public verifiability, and mutual authentication are all factors that may have an impact on it.

#### 4.2.2. Lesson Learned, Discussion, and Open Challenges

Based on our findings from the literature stated above and (Table 4), each technique has its own set of advantages and disadvantages, making it difficult to determine which technique is preferable to the others. We believe that schemes based on ECC would be more efficient than bilinear pairing. Secondly, Amin et al. [80] did not present any formal proof, while the schemes of Anyembe et al. [81] and Gao et al. [82] have been demonstrated to be secure using the ROM. Besides, in terms of security requirements, the scheme presented by Amin et al. [80] has been suffering from forward secrecy, public verifiability, anti-replay attack and mutual authentication. The scheme of Anyembe et al. [81] has the deficiencies of forwarding secrecy and anti-replay attack. The scheme of Gao et al. [82] can be compromised against forward secrecy, public verifiability, mutual authentication and anti-replay attack. However, proposing a novel strategy that is secure in the standard model using ECC remains an unresolved challenge. Additionally, the compact scheme that can achieve all the security requirements is still open.

### 4.3. Hyperelliptic Curve Cryptosystem (HCC) Based WBANs Schemes

HCC is a public cryptography approach that is similar to ECC in that it is an extension of it. When compared to other encryption techniques, such as ECC, RSA, and the Digital Signature Algorithm (DSA), the HCC gives the same level of security. Due to its modest key size, HCC is ideal for resource-constrained situations. The HCC is divided into species of the genus: 2, 3, 4, 5, and 6, with genus 2 being the most secure. The security of HCC is influenced by the hyperelliptic curve discrete logarithm problem, which prohibits an attacker from breaking the keys even if the P and Q are publicly known.

#### 4.3.1. Hyperelliptic Curve Discrete Logarithm Problem (HCDLP)

For the HCDLP, the following complexity assumptions have been made.

Let Ω ∈ {1,2,3,….(y−1)} and W=Ω·D, then finding Ω from W is called HCDLP.

In 2016, Iqbal et al. [83] constructed a new signcryption approach that satisfies the security criteria of public verifiability while remaining cost-effective. As part of this novel method, the authors carry out the Cluster head selection procedure. They claim that the hyperelliptic curve, which is ideal for resource-intensive applications like WBANs. The network model used in this paper, however, was unable to establish a central authority and had problems with certificate renewal and revocation, among others. The authors also fail to explain in any depth the property of public verifiability security, even though the title of this article is largely concerned with this element. Furthermore, there is no consideration for non-repudiation, mutual authentication, or anti-replay attacks in terms of security service.

WBANs are being used to enable the IoT, and Ullah et al. [84] have developed a certificate-based signcryption and energy-efficient access control approach for them. The mathematical structure of HEC is used to determine the cost and security efficiency of the scheme. The authors of the given technique claim that it is more cost-effective and that it provides better security services, such as confidentiality, unforgeability, anti-replay attack, integrity, public verifiability, and forward security, than other techniques currently available. To put it another way, the need for certificate management across an extensive network could affect your overall strategy. Additionally, the absence of mutual authentication and anonymity features may have an impact.

In 2021, Noor et al. [85] presented a new framework for WBANs based on a hyperelliptic curve termed secure channel free certificateless signcryption technique. The authors, on the other hand, were unable to provide any kind of formal or informal proof to support any of the claim security requirements.

#### 4.3.2. Lesson Learned, Discussion, and Open Challenges

Hyperelliptic Curve Cryptosystem (HCC) is one of the most suitable for WBANs with limited resources in terms of power efficiency, memory, and computing and communication capabilities. Based on our findings from the literature stated above and (Table 5), each technique has its own set of advantages and disadvantages, making it difficult to determine which technique is preferable to the others. We believe that schemes based on HCC would be more efficient than bilinear pairing and HCC. Secondly, Iqbal et al. [83], Insaf et al. [84], and Noor et al. [85] did not present any formal proof. Besides, in terms of security requirements, the scheme presented by Iqbal et al. [83] has been suffering from non-repudiation, mutual authentication, or anti-replay attack. The scheme of Insaf et al. [84] has the deficiencies of mutual authentication and anonymity. The scheme of Noor et al. [82] fails to provide the security properties mentioned above. However, proposing a novel strategy that is secure in the standard model or ROM using HCC remains an unresolved challenge. Additionally, the compact scheme that can achieve all the security requirements is still open.

## 5. Comparative Analysis

Throughout this section, we will compare all of the proposed WBANs signcryption schemes based on their computation time, communication overhead, security hardness, security strength, and security properties, among other factors.

### 5.1. Performance Evaluation Matrices

WBANs are distinguished from other networks by the significant hardware limitations they have. WBAN processes should therefore use the least memory and processing power possible while transferring the least amount of data possible utilizing the smallest number of messages to reduce overall energy usage. Performance analysis is typically included in publications since the constraints are so tight. This helps authors illustrate the success of their strategy to tackle the challenge. Often, the costs associated with computation, communication, and energy are separated out and included in the analysis [50].

#### 5.1.1. Computation Time

Computation time is the most essential performance indicator. As the sensor nodes do not have much processing capacity, and because additional computing uses up more of the very limited energy supply, schemes must be as computationally efficient as possible. The most frequent way for calculating computation cost is to time how long it takes for the necessary processes to complete: [69,70,71,72,73,74,75,76,77,78,79,80,81,82,83,84,85]. The times are frequently compared to those of other schemes to provide some further meaning to the observed time [65,66,67,68,69,70,71,72,73,74,75,76,77,78,79]. Different approaches to analyzing the computing cost emerge when compared to other schemes. One of the most frequent approaches is to count the number of distinct operations that must be performed (e.g., pairing operations, exponentiation, etc.) and then compare the results to those of other schemes [69,70,71,72,73,74,75,76,77,78,79,80,81,82,83,84,85].

For [69,70,71,72,73,74,75,76,77,78,79,80,81,82,83,84,85], we use the same performance criteria as in [86] to provide a quantitative study of communication overhead and computing cost. Table 6 shows the values obtained from [86], which include exponentiation, pairing operation, pairing-based scalar point multiplication, Elliptic curve-based point multiplication, and Hyperelliptic Curve Divisor Multiplication. According to the experimental results discussed in [86], a pairing operation consumes 20.04 ms, an exponentiation operation takes 5.31 ms, elliptic curve scalar point multiplication takes 2.21 ms. According to [87,88,89,90], hyperelliptic curve devisor multiplication takes half the time as compared to ECC, so it takes 1.105 ms, respectively. Thus, based on computing time and communication overhead, we can simply choose the optimum scheme from Table 7, Table 8 and Table 9.

**Signcryption Phase**

**Figure 9:**
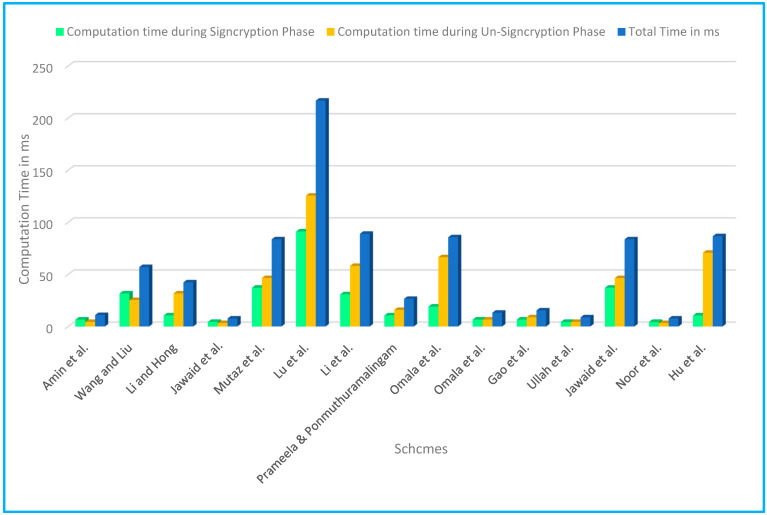
Computation Time of Signcryption and Un-signcryption Phase.

For signcryption algorithm, the scheme of Amin et al. [80] requires three SPMEC operations, Wang and Liu [69] scheme need one BPM, one P and one EXP operation, Li and Hong [70] two EXP, Jawaid et al. [83] require four HCDM, Mutaz et al. [71] requires one EXP and five BPM, Lu et al. [72] two BPM, eleven EXP, one P operations, Li et al. [73] needs four BPM, and one EXP operations, Prameela & Ponmuthuramalingam [74] requires two EXP, Omala et al. [75] requires three BPM, Omala et al. [81] three SPMEC, Gao et al. [82] requires three SPMEC, Ullah et al. [84] requires four HCDM Jawaid et al. [76] require five BPM, and one EXP, Noor et al. [85] requires four ℋCDℳ whereas the scheme of Hu et al. [77] requires two EXP operations respectively. Furthermore, Table 7 and Figure 9 illustrate a comparison of main cryptographic operations utilized in the signcryption phase of the proposed schemes suggested for WBANs while Table 8 shows the comparison of major operations in terms of milliseconds.

**Un-Signcryption Phase**

For the Un-Signcryption algorithm, the scheme of Amin et al. [80] requires two SPMEC operations, Wang and Liu [69] scheme needs one P and one EXP operations, Li and Hong [70] require one EXP, one P, and one BPM Jawaid et al. [83] requires three HCDM, Mutaz et al. [71] requires two P and one BPM, Lu et al. [72] requires one EXP, and six P operations, Li et al. [73] needs two BPM, one EXP, and two P operations, Prameela & Ponmuthuramalingam [74] requires three EXP, Omala et al. [75] requires one BPM and three P operations, Omala et al. [74] four SPMEC, Gao et al. [82] requires four SPMEC, Ullah et al. [84] requires four HCDM Jawaid et al. [76] require one BPM, and two P, Noor et al. [85] requires three HCDM whereas the scheme of Hu et al. [77] requires two EXP and two P operations, respectively. Furthermore, Table 9 and Figure 9 illustrate a comparison of main cryptographic operations utilized in the un-signcryption phase of the proposed schemes suggested for WBANs while Table 10 shows the comparison of major operations in terms of milliseconds.

The number of expensive operations required for the signcryption and un-signcryption processes is used to calculate the computational time. This cost represents the amount of computing effort required by both the sender and the recipient of the signed communication. Multiplication and exponentiation are common examples of these operations. In terms of hardware implementation, the number of these operations determines the computational time [86].

#### 5.1.2. Security Hardness

In this part, we analyze the security of the suggested signcryption techniques presented for securing WBANs through quantitative analysis, including security attributes. Table 11 provides a security comparison of [69,70,71,72,73,74,75,76,77,78,79,80,81,82,83,84,85]. The √ represents this security attribute is satisfied.

#### 5.1.3. Security Strength

Security verification is very important in analyzing the security properties of cryptographic schemes and can be used to prove their correctness also. It is critical to ensure that the security necessities/requirements are satisfied. Normally, a ROM or Standard Model is used to assess the security strength of signcryption techniques. The √ represents this security strength is satisfied as shown in Table 12.

#### 5.1.4. Communication Overhead

Communication overhead measurement is critical since it is the most energy-intensive of all operations. The amount of the sent data or the number of messages sent is the most typical approach of measuring the communication cost, as seen in [69,70,71,72,73,74,75,76,77,78,79,80,81,82,83,84,85]. The authors, as before, want to put their findings in context by comparing them to other methods. In [69,70,71,72,73,74,75,76,77,78,79,80,81,82,83,84,85], the number of bits conveyed was compared, as indicated in Table 13.

**Figure 10:**
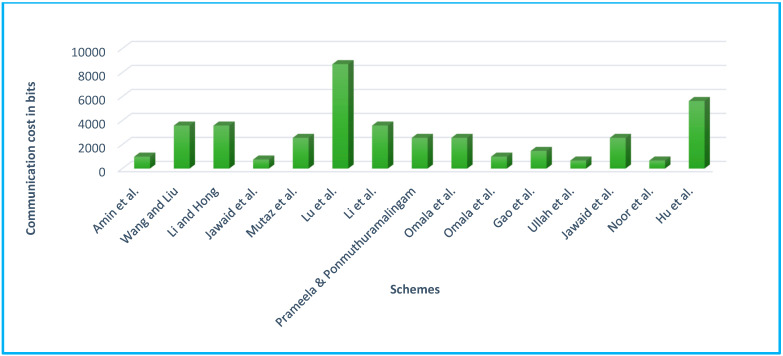
Communication Overhead of all the Signcryption Schemes Presented for WBANs.

Furthermore, according to [87,88,89,90], bilinear pairing (|*G*|), ECC (|*q*|), and hyperelliptic curve (|*n*|) use 1024 bits, 160 bits, and 80 bits key sizes, and message |*m*| = 512 bits, respectively, for communication overhead. We may conclude that the HCC will be the most cost-effective alternative in terms of communication overhead for WBANs with low bandwidth capacity of the type described above, as shown in Table 14 and Figure 10.

#### 5.1.5. Lessen Learned and Discussion

The most ideal method of evaluating performance is to employ methods that are not reliant on external sources. The length of time required for the process and the number of operations required were the two most commonly used criteria for calculating computational cost. Unfortunately, neither of them is without flaws. There is a significant impact on time measurements due to the performance of the device to which the method is applied. When comparing schemes, the number of operations is the most advantageous choice because it reduces the reliance on other elements of the plan. When comparing the implementation of individual schemes, however, it is necessary to use the same algorithms in all of the schemes under consideration. The quantity of data transferred, and the number of messages sent and received are the two metrics that are most commonly used in communication cost analysis. Both of these indicators are significant, but they are distinct from one another in their significance. A useful indicator is undoubtedly the size of the transmitted data because sending more data consumes more energy. However, sending several smaller messages is significantly more expensive than sending a single large message because they incur significantly more overhead. Therefore, it is probably best to incorporate both measures into your plan as much as you can whenever possible. Authors rarely do this, as evidenced by the survey results.

### 5.2. Evaluation Based on Distance from Average Solution (EDAS)

Ghorabaee et al. [91] offer the EDAS technique, which ranks given schemes based on the average solution obtained. The average solution is derived by computing the Positive Distance from Average and the Negative Distance from Average. It is generally agreed that the scheme with the highest values is the highest-ranked scheme [92]. In the fuzzy-EDAS approach, the alternatives are ranked according to the decreasing value of the defuzzified appraisal score [93], which is obtained from the defuzzified appraisal score. Table 15 shows the criterion that was used to rank the schemes based on their assessment score and how it was determined. A more in-depth description of the phases involved in the fuzzy-EDAS technique is provided in the subsequent section.

The following section outlines the phases involved in applying fuzzy-EDAS approach to a decision making situation.

Step-1:

Table 15 above shows the equations used to derive the weights for the prior related schemes, which are applied to the selected matrices.

Step-2:

According to Table 16, the following Equations and Table 15 are utilized to build a fuzzy average decision matrix with regard to all of the relevant matrices:

(1) $$\left(\phi \right)={\left[\vartheta_{b}\right]}_{1\times \beta },$$

While

(2) $$=\frac{\sum_{i=1}^{y}\;X_{ab}}{y}$$

Step-3:

This phase of the fuzzy-EDAS approach uses these equations to compute the matrices for fuzzy Positive Distance from Average (PDA) and fuzzy Negative Distance from Average (NDA), as shown in Table 17 and Table 18.

(3) $$P_{av}={\left[{\left(P_{av}\right)}_{ab}\right]}_{\beta \times \beta }$$

If the state bth is favorable, then

(4) $${\left(P_{av}\right)}_{ab}=\frac{max\left(0,\left(Ave_{b}-X_{ab}\right)\right)}{Ave_{b}}$$

And for less favorable, it becomes;

(5) $${\left(P_{av}\right)}_{ab}=\frac{max\left(0,\left(X_{ab}-Ave_{b}\right)\right)}{Ave_{b}}$$

(6) $$\left(N_{av}\right)={\left[{\left(N_{av}\right)}_{ab}\right]}_{\beta \times \beta }$$

If the bth criterion is more favorable than

(7) $${\left(N_{av}\right)}_{ab}=\frac{max\left(0,\left(Ave_{b}-X_{ab}\right)\right)}{Ave_{b}}$$

And less desirable, then the given above equations become

(8) $${\left(N_{av}\right)}_{ab}=\frac{max\left(0,\left(X_{ab}-Ave_{b}\right)\right)}{Ave_{b}}$$

Step-4:

During this step, the fuzzy-weighted positive and negative distance matrices are generated, as illustrated by the examples in Table 19 and Table 20. This is accomplished through the use of the equations listed below.


(9) $${WP}_{av}\;=\;\sum_{b=1}^{y}\;\lambda_{b}{\left(\mathrm{PD}\right)}_{ab}$$

(10) $${WN}_{av}\;=\;\sum_{b=1}^{y}\;\lambda_{b}{\left(\mathrm{ND}\right)}_{ab}$$

Step-5:

The fuzzy evaluation score for various alternatives is determined in the penultimate step by utilizing the following equations, which are given below. Among the selected schemes, the alternative schemes with the greatest value of the assessment score are the best, as shown in Table 21, and they are the ones that should be pursued.

(11) $$N\left({WP}_{av}\right)=\frac{{WP}_{av}}{{max}_{a}\left({WP}_{av}\right)}$$

(12) $$\begin{array}{c} N(WN_{av})=1-\frac{WN_{av}}{{max}_{a}\left(WN_{av}\right)} \end{array}$$

(13) $$M=\frac{1}{2}\left(NWSPD_{avg}-{NWN}_{av}\right)$$

where 0 ≤M≥ 1.

In this section, the methodology described above is applied to the solution of a case study on the selection of various efficient schemes such as Amin et al. [80], Wang and Liu [69], Li and Hong [70], Jawaid et al. [83], Mutaz et al. [71], Lu et al. [72], Li et al. [73], Prameela & Ponmuthuramalingam [74], Omala et al. [75], Omala et al. [81], Gao et al. [82], Ullah et al. [84], Jawaid et al. [76], Noor et al. [85] and Hu et al. [77].

All other criteria, with the exception of communication overhead and computational cost, are unfavorable. By combining Equations (1) and (2), we were able to calculate the objective weights for all of the decision matrices that had been collected from the three decision-makers. Finally, aggregate weights were generated by multiplying the sum of all objective weights for each criterion by 100. Table 15 summarizes the individual objective weights for each condition as well as the aggregated objective weights. After that, an average decision matrix was built, the results of which are displayed in Table 16. As indicated in Table 15, the average result was derived by applying Equations (3)–(8) to the entire number of solutions created, which includes the average solution;s crisp value. The positive and negative distances from the average values were calculated using Equations (9) and (10), and the results are displayed in Table 18 and Table 19. Equations (11) and (12) are utilized to generate the fuzzy appraisal score for various options based on their fuzzy assessment scores in the penultimate stage. To finish up, Equation (12) was employed in order to rank the alternatives in accordance with the defuzzified appraisal score. Table 21 shows a visual representation of all of these values. The Noor et al. [85] scheme was found to be the most effective alternative solution for a WBANs system.

#### Lesson Learned

The EDAS technique was used to analyze the suggested WBANs domain signcryption to discover the idlest solution among them. Signcryption and Un-Signcryption Time, Communication Overhead, Security Hardness, Security Strength, and Security Requirement are the performance metrics we use for this. According to the results, the solution proposed by Noor et al. [85] outperforms the proposed methods in the area of WBANs.

The approach proposes by Noor et al. [85] outperforms the remaining WBANs domain solutions. However, in terms of security requirements and security strength, this system should be improved. The approach proposed by Noor et al. [85] is not supported by any computational model, including the Standard Model/ROM. As a result, under the standard computation paradigm, a secure HCC-based secure technique is required.

## 6. Conclusions, Discussion, and Future Work

Signcryption is a critical factor of secure communication; it is the first step toward secure communication and assists networks in decreasing unwanted users and avoiding deceptions. Until now, no survey has conducted an in-depth examination of secure signcryption procedures in WBANs; the proposed study is the only one that does so, and it may be of interest to readers and new researchers in this specialized field. In the table forms, we have displayed useful information or features of several signcryption techniques. In addition to the tables, we have developed numerous diagrams to show the architecture, taxonomy, and efficiency analysis of all (to the best of our knowledge) signcryption schemes covered in this survey, in addition to the tables. The survey starts with some basic information about WBANs, such as architecture, applications and security requirements. These details are crucial for new readers to gain a better understanding of WBANs architecture, while also assisting different designers in the development of various signcryption schemes. According to our survey, WBANs signcryption schemes are classified as Attribute-based signcryption, Identity-based signcryption, PKI-based signcryption, Certificateless signcryption, Certificate-based signcryption, and Heterogeneous signcryption techniques. This survey also explains all the secure signcryption schemes in WBANs, divides them into categories depending on the hardness algorithm utilized, and describes each hardness methodology in depth. A full explanation is drawn at the end of the section, which illustrates various aspects of each scheme based on the hardness algorithm, security properties, and strength of the schemes.

Finally, the survey completes with a conclusion and future directions section, which not only draws a few findings but also identifies several important research areas that should be investigated shortly. As WBANs are one of the most promising developing technologies in the field of E-health, and shortly, they will fundamentally revolutionize people’s healthcare systems by providing a plethora of services and freeing them from the need to attend traditional hospitals. Apart from its importance in the realm of E-health, WBANs face numerous security risks as a result of wireless communication. Signcryption is an increasingly essential problem about secure communication in WBANs, thus it is critical to have safe signcryption solutions; these help the network reduce unwanted users and protect them from illegal activities.

Notably, the solutions offered in the literature for securing the WBANs environment are not efficient in some aspects, they fall short of meeting the necessary requirements for security. As the solutions based on certificateless cryptography that have been adopted for WBANs are generally hampered by the distribution of partial keys. In contrast to the solutions based on Identity-Based Cryptography, which can be affected from key escrow while Certificate-Based Cryptography are not suitable for large numbers of users.

WBAN solutions are frequently utilised in data-intensive applications where patients generate large volumes of data. The data is saved on a cloud server where machine learning tools extract, prepare, and analyse it. The algorithm takes a few days to several months to process. Important issues to consider when using this method include security issues.

The majority of the devices that are used in the WBANs domain are limited in terms of resources. These devices are limited in terms of computational power and storage capacity. As obvious from our survey, the signcryption solutions that have been implemented for WBANs are time-consuming. The solutions that are currently available were constructed using asymmetric algorithms such as bilinear pairing and ECC. According to Hussain et al. [94], ECC and Bilinear Pairing are unsuitable for resource-limited technologies due to their high energy consumption. An alternative cryptographic algorithm such as the HCC or the Chebyshev chaotic map should be implemented to achieve a better balance between energy consumption and security strength. Ideally, the signcryption solution should be able to provide appropriate security while consuming minimum energy on the resource-constrained devices of WBANs.

In this survey, we discussed analysed all the existing signcryption schemes proposed for WBANs. However, there is no signcryption method or scheme that can guarantee perfect communication security. Designing a secure WBANs signcryption system necessitates an appropriate mapping of signcryption methods or schemes with various signcryption parameters. We analyzed numerous signcryption methods in this survey study, divided them based on the security hardness algorithm utilized, and highlighted their benefits, drawbacks, limitations, and resilience against various security threats; these may be useful for enhancing the signcryption process in WBANs. However, additional effort is required to design a novel signcryption scheme that meets the stringent secure communication requirements of WBANs applications. The multi-criteria decision-making approach is used for a comparative examination of the existing signcryption schemes. Since WBANs are still in their infancy, they face several challenges. As a result, it is critical to implement effective solutions to address these difficulties. Secure signcryption has recently emerged as one of the major issues in this sector, and more effort will be necessary in the future to address this issue. As technology improves, it is becoming more challenging to construct lightweight secure signcryption mechanisms for devices with constrained resources.

The challenges of security for WBANs are discussed in this study. Due to the sensitivity of the sensor messages being transferred to and from the human body, the WBANs technology places a premium on security. We identify many key security requirements for Signcryption, which are essential for assuring security in WBANs. It is important to analyse the strengths and weaknesses of all signcryption schemes, as well as their compliance with security standards, attack resistance, and overall performance. To aid researchers and developers in identifying and distinguishing essential aspects of WBAN security, the security and efficiency of existing WBANs Signcryption methods are reviewed. For those working on unique security solutions for WBANs, we hope that this work will serve as a guide and a reference in the future.

Future research will need to improve existing signcryption approaches, as well as propose a new WBANs scheme based on maintaining a trade-off between efficiency and security. There may be a need for increased adaptability and interoperability with sensing equipment from different vendors when developing a secure WBAN signcryption solution.

Due to intensive pairing processes, most of the authors’ use pairing-based cryptography, which is inefficient notably in the implementation of WBANs. Hence, developing an effective WBAN signcryption technique is a task that remains unsolved.

It is necessary to investigate the security proofs of existing solutions in order to demonstrate the security of WBANs not only in the ROM but also in the standard computational model. Unfortunately, none of the existing’s solutions are proven under the standard computational model.

To improve the approach taken by Noor et al. [85], which does not involve the use of a secure channel for the distribution of partial keys among the entities, additional work must be done. Even though the authors did not give any formal or informal evidence. The solution of Noor et al. [85] needs to be further polished with the assumption of HCDLP under the standard computational model. Because of its minimal key size and compact security, the HCDLP should be properly considered when constructing secure WBANs-based signcryption solutions using a standard computational model.

Lightweight secured schemes that are easy to manage will be required in the future for intelligent environments like smart homes, particularly in the field of WBANs, to manage security and provide quick responses to users. Another requirement is to develop signcryption methods that provide a better trade-off between energy consumption and security strength, which can be accomplished by reducing the complexity of the schemes used in the signcryption process. To sum it up, there are still many challenges to overcome on the road to developing an unobtrusive, user-friendly, and secure WBANs system. Additionally, there are numerous new research directions in WBANs that must be investigated as soon as possible.

## Figures and Tables

**Table 1:** Summary of the Related Surveys.

Authors and Ref. No.	Publication Year	Findings
Saleem et al. [32]	2009	Highlight main security requirements and DDoS concerns Provide a thorough assessment of existing security protocols for WBANs
Zhang et al. [33]	2011	Investigate probable resource-constrained WBANs attacks Present state-of-the-art communication protocols, cryptographic algorithms, and key management strategies Examine existing solutions’ flaws and probable future research areas in WBANs
Aqeel et al. [34]	2013	Offer a critical analysis of potential WBANs authentication techniques in the light of IEEE 802.15.6 standard
Javadi and Razzaque [35]	2013	Examine major security and privacy issues as well as potential threats Discuss an unsolved Quality of Service (QoS) problem in WBANs Outline future directions
Saha and Anvekar [36]	2014	Present a state-of-the-art of existing WBANs security aspects. Highlights several significant security challenges
Pathania and Bilandi [37]	2014	Outline of WBANs and related challenges from a security perspective Discuss security attacks and security necessities in WBANs
Kang and Adibi [38]	2015	Investigate the security features of application and communication protocols Discuss the architecture, vulnerabilities, and attacks, as well as future opportunities of WBANs
Mainanwal et al. [39]	2015	Summarise the benefits and drawbacks of different security and privacy solutions used in WBANs Outline future directions
Usha and Priya [40]	2015	Address various types of attacks, prevention strategies, and simulation tools for WBANs
Masdari and Ahmadzadeh [41]	2016	Conduct a comprehensive review and analysis of the numerous authentication schemes presented in WBANs Discuss the benefits and drawbacks of various authentication techniques Outline future directions
Naik and Samundiswary [42]	2016	Present an overview of WBANs and WSNs Discuss WBANs security protocols with their advantages and disadvantages
Al-Janabi et al. [43]	2017	Examine the communication architecture of WBANs, as well as their security and privacy needs, security threats, and important issues. Outline future directions
Sawaneh et al. [44]	2017	Focuses on building and implementing WBANs in healthcare systems Provide a brief overview of WBAN security and privacy requirements
Zou et al. [45]	2017	Examine a wide range of secure communication solutions within WBANs and between external entities Emphasizes the importance of primary security requirements for secure transmission at both levels
Aman and Shah [46]	2017	Conduct a thorough review of significant studies on mobile, ubiquitous, and WBANs, focusing on routing and security challenges
Narwal and Mohapatra [47]	2018	Provide a comprehensive analysis of several authentication approaches Add a complete analysis of the schemes based on security attacks, security features, and a variety of other factors
Usman et al. [48]	2018	Investigate Security issues at all WBANs layers Underline future directions
Malik et al. [49]	2018	Present a broad overview of major security requirements and potential attacks in WBANs at various layers of the OSI model
Kompara and Holbl [50]	2018	A comprehensive overview of existing key agreement methods is presented, with each method being divided into four categories
Morales et al. [51]	2019	Aims to provide a holistic security picture of the entire WBANs system
Bharathi and Venkateswari [52]	2019	Present an overview of WBANs, their applications, and security concerns
Nidhya and Karthk [53]	2019	Review the security and privacy issues of electronic healthcare record systems in WBANs
Joshi and Mahopatra [54].	2019	Analyze Authentication protocols design issues in WBANs
Chaudhary et al. [55]	2019	Explore the security and privacy difficulties with WBANs Describe the type of authentication technique that can be employed at a particular stage.
Hussain et al. [56]	2019	Provide an overview of WBANs and their properties Compares various authentication techniques, highlighting their advantages, disadvantages, performance evaluation, and robustness against various security attacks Outline future directions
Asam et al. [57]	2019	Present a thorough assessment of the issues in WBANs from the perspectives of communication and security
Karchowdhury and Sen [58]	2019	Discuss security requirements and Denial of Service concerns
Roy et al. [59]	2020	Present a comprehensive analysis on WSNs and WBANs security and privacy challenges Examine the characteristics, architecture, performance measures, and applications of both in-depth, and then conduct a comparison analysis Outline future research direction
Sharma and Kang [60]	2020	Examine and evaluate WBANs routing, security, energy, and cost-cutting problems
Hajar et al. [61]	2021	Overview WBANs technology with a special focus on security and privacy concerns and countermeasures Outline future research directions
Vignesh and Sivakumar [62]	2021	Discuss security and routing issues that WBANs face with a preventative mechanism that is in place.
Jabeen et al. [63]	2021	Review different security approaches of WBANs Investigate the feasibility of multiple attacks while keeping memory restrictions in mind
Narwal and Mahopatra [64]	2021	Discuss various security and authentication schemes and solutions Discuss WBANs applications, open research issues, recommendations, and future trends
Proposed	2021	Surveys all the WBANs signcryption schemes and compared based on EDAS technique to show the efficiency of each. Furthermore, the study emphasized the security issues that the previously suggested schemes face, as well as future work for WBANs.

**Table 2:** Summary of the qualitative comparison of the existing surveys with the proposed survey.

Authors and Ref. No.	One	Two	Three	Four	Five	Six	Seven	Eight
Saleem et al. [32]	√	×	×	√	×	×	×	×
Zhang et al. [33]	√	×	×	√	×	×	×	×
Aqeel et al. [34]	√	×	×	√	×	√	×	×
Javadi and Razzaque [35]	√	×	×	√	×	√	×	√
Saha and Anvekar [36]	√	×	×	√	×	√	×	×
Pathania and Bilandi [37]	√	×	×	√	×	×	×	×
Kang and Adibi [38]	√	×	×	√	×	×	×	×
Mainanwal et al. [39]	√	×	×	√	×	×	×	×
Usha and Priya [40]	√	×	×	√	×	×	×	×
Masdari and Ahmadzadeh [41]	√	×	√	√	×	√	×	×
Naik and Samundiswary [42]	√	×	√	√	×	×	×	×
Al-Janabi et al. [43]	√	×	×	√	×	√	×	×
Sawaneh et al. [44]	×	×	×	√	×	×	×	×
Zou et al. [45]	√	×	√	√	×	√	×	√
Aman and Shah [46]	√	×	√	×	×	√	×	×
Narwal and Mohapatra [47]	√	×	√	√	×	×	×	×
Usman et al. [48]	√	×	×	√	×	√	×	×
Malik et al. [49]	√	×	√	√	×	×	×	×
Kompara and Holbl [50]	√	×	√	√	√	√	×	×
Morales et al. [51]	√	×	×	√	×	√	×	×
Bharathi and Venkateswari [52]	√	×	×	√	×	×	×	×
Nidhya and Karthk [53]	√	×	√	√	×	×	×	×
Joshi and Mahopatra [54].	×	×	√	√	×	√	×	√
Chaudhary et al. [55]	√	×	√	√	×	×	×	×
Hussain et al. [56]	√	×	√	√	×	√	×	√
Asam et al. [57]	√	×	×	√	×	×	×	√
Karchowdhury and Sen [58]	√	×	×	√	×	×	×	×
Roy et al. [59]	√	×	×	√	×	√	×	√
Sharma and Kang [60]	√	×	×	√	×	×	×	×
Hajar et al. [61]	√	×	√	√	×	√	√	√
Vignesh and Sivakumar [62]	√	×	×	√	×	×	×	×
Jabeen et al. [63]	√	×	√	√	×	√	√	×
Narwal and Mahopatra [64]	√	×	√	√	√	×	√	√
Proposed	√	√	√	√	√	√	√	√

One: WBANs Architecture, Two: Signcryption schemes consideration, Three: Limitation and strength of WBANs security solutions, Four: Security Requirements, Five: Performance analysis, Six: Open Research Directions and future suggestion, Seven: Comparison with existing’s Surveys, Eight: WBANs applications, √ demonstrate a specific area covered, × demonstrate a survey lake a specific area.

**Table 3:** Limitations of Bilinear Pairing based Signcryption Schemes presented for securing WBANs.

Authors and Ref. No.	Publication Year	Limitations
Wang and Liu [69]	2015	Fails to address the key escrow issue High computing power consumption and increased nature of communication bandwidth due to bilinear pairing
Li and Hong [70]	2016	Affected by a partial distribution of private keys Utilize bilinear pairing for security hardness which is a computationally intensive operation
Mutaz et al. [71]	2018	Affected by a partial distribution of private keys Affected by certificate related issues such as certificate distributions, certificate revocation, and certificate administration Utilize bilinear pairing for security hardness which is a computationally intensive operation
Lu et al. [72]	2018	The scheme may experience issues with private key distribution and key escrow due to the use of the PKG principle Utilize bilinear pairing for security hardness which is a computationally intensive operation
Li et al. [73]	2018	Affected by a partial distribution of private keys Utilize bilinear pairing for security hardness which is a computationally intensive operation
Prameela and Ponmuthuramalingam [74]	2018	Affected by a partial distribution of private keys Use bilinear pairing for security hardness which is a computationally intensive operation
Anyembe et al. [75]	2018	Affected by a partial distribution of private keys Affected by certificate related issues such as certificate distributions, certificate revocation, and certificate administration Utilize bilinear pairing for security hardness which is a computationally intensive operation
Iqbal et al. [76]	2019	Affected by a partial distribution of private keys Affected by certificate related issues such as certificate distributions, certificate revocation, and certificate administration Utilize bilinear pairing for security hardness which is a computationally intensive operation
Hu et al. [77]	2021	Affected from key escrow problem of identity-based cryptography Affected by certificate related issues such as certificate distributions, certificate revocation, and certificate administration Utilize bilinear pairing for security hardness which is a computationally intensive operation

**Table 4:** Limitations of ECC based Signcryption Schemes presented for securing WBANs.

Authors and Ref. No.	Publication Year	Limitations
Amin et al. [80]	2014	Affected by certificate related issues such as certificate distributions, certificate revocation, and certificate administration Use ECC with a key size of 160 bits which may incur high computing power consumption and increase bandwidth
Anyembe et al. [81]	2018	Hamper by the requirement of a secure channel for distribution of partial keys from the application provider’s and the controller’s key escrow problem Use ECC with a key size of 160 bits which may incur high computing power consumption and increase bandwidth
Gao et al. [82]	2019	Hamper by the requirement of a secure channel for the distribution of partial private key Use ECC with a key size of 160 bits which may incur high computing power consumption and increase bandwidth

**Table 5:** Limitations of Hyperelliptic Curve based Signcryption Schemes presented for securing WBANs.

Authors and Ref. No.	Publication Year	Limitations
Iqbal et al. [83]	2016	Fail to establish a central authority and had issues with certificate distributions, certificate revocation, and certificate administration Unable to provide formal proof in either ROM/Standard Model
Insaf et al. [84]	2019	Necessitating certificate management in a network with a high number of devices might have an impact Unable to provide formal proof in either ROM/Standard Model
Noor et al. [85]	2021	The authors made a false claim by claiming the security requirements of confidentiality, forward secrecy, anonymity, and anti-replay attack. Unable to provide formal proof in either ROM/Standard Model/informal

**Table 6:** Computation Time of Costly Mathematical Operations in Milliseconds.

Descriptions	Operation Time in Milliseconds
Exponentiation (EXP)	5.31
Pairing Operation (P)	20.04
Pairing based scalar point multiplication (BPM)	6.38
Elliptic curve based point multiplication (SPMEC)	2.21
Hyperelliptic Curve Divisor Multiplication (HCDM)	1.105

**Table 7:** Cryptographic Operations Utilised in the Signcryption Phase.

Authors and Ref. No.	Signcryption
Amin et al. [80]	3 SPMEC
Wang and Liu [69]	1 EXP +1 BPM +1 P
Li and Hong [70]	2 EXP
Jawaid et al. [83]	4 HCDM
Mutaz et al. [71]	5 BPM +1 EXP
Lu et al. [72]	2 BPM +11 EXP +1 P
Li et al. [73]	4 BPM +1 EXP
Prameela & Ponmuthuramalingam [74]	2 EXP
Omala et al. [75]	3 BPM
Omala et al. [81]	3 SPMEC
Gao et al. [82]	3 SPMEC
Ullah et al. [84]	4 HCDM
Jawaid et al. [76]	5 BPM +1 EXP
Noor et al. [85]	4 HCDM
Hu et al. [77]	2 EXP

**Table 8:** Computation Time in Milliseconds (Signcryption Phase).

Authors and Ref. No.	Computation Time during Signcryption Phase
Amin et al. [80]	6.63
Wang and Liu [69]	31.73
Li and Hong [70]	10.62
Jawaid et al. [83]	4.42
Mutaz et al. [71]	37.21
Lu et al. [72]	91.21
Li et al. [73]	30.83
Prameela & Ponmuthuramalingam [74]	10.62
Omala et al. [75]	19.14
Omala et al. [81]	6.63
Gao et al. [82]	6.63
Ullah et al. [84]	4.42
Jawaid et al. [76]	37.21
Noor et al. [85]	4.42
Hu et al. [77]	10.62

**Table 9:** Cryptographic Operations Utilised in the Un-Signcryption Phase.

Authors and Ref. No.	Unsigncryption
Amin et al. [80]	2 SPMEC
Wang and Liu [69]	1 EXP + 1 P
Li and Hong [70]	1 P + 1EXP + 1BPM
Jawaid et al. [83]	3 ℋCDℳ
Mutaz et al. [71]	1 BPM +2 P
Lu et al. [72]	6 P+1 EXP
Li et al. [73]	2 BPM +1 EXP +2 P
Prameela & Ponmuthuramalingam [74]	3 EXP
Omala et al. [75]	1 BPM +3 P
Omala et al. [81]	3 SPMEC
Gao et al. [82]	4 SPMEC
Ullah et al. [84]	4 ℋCDℳ
Jawaid et al. [76]	1 BPM + 2 P
Noor et al. [85]	3 ℋCDℳ
Hu et al. [77]	3 P + 2 EXP

**Table 10:** Computation Time in Milliseconds (Un-Signcryption Phase).

Authors and Ref. No.	Computation Time during Un-Signcryption Phase
Amin et al. [80]	4.42
Wang and Liu [69]	25.35
Li and Hong [70]	31.73
Jawaid et al. [83]	3.315
Mutaz et al. [71]	46.46
Lu et al. [72]	125.55
Li et al. [73]	58.15
Prameela & Ponmuthuramalingam [74]	15.93
Omala et al. [75]	66.5
Omala et al. [81]	6.63
Gao et al. [82]	8.84
Ullah et al. [84]	4.42
Jawaid et al. [76]	46.46
Noor et al. [85]	3.315
Hu et al. [77]	70.74

**Table 11:** Comparative Analysis of WBANs Signcryption Schemes based on Security Hardness.

Ref. No.	Bilinear Pairing	ECC	HEC
Amin et al. [80]		√	
Wang and Liu [69]	√		
Li and Hong [70]	√		
Jawaid et al. [83]			√
Mutaz et al. [71]	√		
Lu et al. [72]	√		
Li et al. [73]	√		
Prameela & Ponmuthuramalingam [74]	√		
Omala et al. [75]	√		
Omala et al. [81]		√	
Gao et al. [82]		√	
Ullah et al. [84]			√
Jawaid et al. [76]	√		
Noor et al. [85]			√
Hu et al. [77]	√		

**Table 12:** Strength based Comparative Analysis of WBANs Signcryption Schemes.

Ref. No.	ROM	N/A
Amin et al. [80]		√
Wang and Liu [69]		√
Li and Hong [70]		√
Jawaid et al. [83]		√
Mutaz et al. [71]	√	
Lu et al. [72]	√	
Li et al. [73]	√	
Prameela & Ponmuthuramalingam [74]	√	
Omala et al. [75]	√	
Omala et al. [81]	√	
Gao et al. [82]	√	
Ullah et al. [84]		√
Jawaid et al. [76]	√	
Noor et al. [85]		√
Hu et al. [77]	√	

**Table 13:** Communication Overhead in terms of major operations of the signcryption in presented for WBANs.

Authors & Ref. No.	Ciphertext Size
Amin et al. [80]	3|q|+|m|
Wang and Liu [69]	3|G|+|m|
Li and Hong [70]	3|G|+|m|
Jawaid et al. [83]	3|q|+|m|
Mutaz et al. [71]	2|G|+|m|
Lu et al. [72]	8|G|+|m|
Li et al. [73]	3|G|+|m|
Prameela & Ponmuthuramalingam [74]	2|G|+|m|
Omala et al. [75]	2|G|+|m|
Omala et al. [81]	3|q|+|m|
Gao et al. [82]	6|q|+|m|
Ullah et al. [84]	2|n|+|m|
Jawaid et al. [76]	2|G|+|m|
Noor et al. [85]	2|n|+|m|
Hu et al. [77]	5|G|+|m|

**Table 14:** Communication Overhead of the Signcryption in Presented for WBANs.

Authors & Ref. No.	Ciphertext Size
Amin et al. [80]	992
Wang and Liu [69]	3584
Li and Hong [70]	3584
Jawaid et al. [83]	752
Mutaz et al. [71]	2560
Lu et al. [72]	8704
Li et al. [73]	3584
Prameela & Ponmuthuramalingam [74]	2560
Omala et al. [75]	2560
Omala et al. [81]	992
Gao et al. [82]	1472
Ullah et al. [84]	672
Jawaid et al. [76]	2560
Noor et al. [85]	672
Hu et al. [77]	5632

**Table 15:** Selected Parameters for EDAS.

Criteria	Non-Beneficial		Beneficial		
Probability	0.2	0.2	0.2	0.2	0.2
Authors and Ref. No.	Computation Time	Communication Overhead	Security Strength	Security Hardness	Security Requirements
Amin et al. [80]	11.05	992	0	0.5	0
Wang and Liu [69]	57.08	3584	0	0	0.5
Li and Hong [70]	42.35	3584	0	0	0
Jawaid et al. [83]	7.735	752	0	1	0
Mutaz et al. [71]	83.67	2560	1	0	1
Lu et al. [72]	216.76	8704	1	0	1
Li et al. [73]	88.98	3584	1	0	1
Prameela & Ponmuthuramalingam [74]	26.55	2560	1	0	1
Omala et al. [75]	85.64	2560	1	0	1
Omala et al. [81]	13.26	992	1	0.5	1
Gao et al. [82]	15.47	1472	1	0.5	1
Ullah et al. [84]	8.84	672	0	1	0
Jawaid et al. [76]	83.67	2560	1	0	1
Noor et al. [85]	7.735	672	0	1	0
Hu et al. [77]	86.67	5632	1	0	1

**Table 16:** Selected Parameters Average.

Authors and Ref. No.	Computation Time	Communication Overhead	Security Strength	Security Hardness	Security Requirements
Amin et al. [80]	11.05	992	0	0.5	0
Wang and Liu [69]	57.08	3584	0	0	0.5
Li and Hong [70]	42.35	3584	0	0	0
Jawaid et al. [83]	7.735	752	0	1	0
Mutaz et al. [71]	83.67	2560	1	0	1
Lu et al. [72]	216.76	8704	1	0	1
Li et al. [73]	88.98	3584	1	0	1
Prameela & Ponmuthuramalingam [74]	26.55	2560	1	0	1
Omala et al. [75]	85.64	2560	1	0	1
Omala et al. [81]	13.26	992	1	0.5	1
Gao et al. [82]	15.47	1472	1	0.5	1
Ullah et al. [84]	8.84	672	0	1	0
Jawaid et al. [76]	83.67	2560	1	0	1
Noor et al. [85]	7.735	672	0	1	0
Hu et al. [77]	86.67	5632	1	0	1
Average	55.69733333	2725.333333	0.6	0.3	0.633333333

**Table 17:** Positive Distance from Average.

Authors and Ref. No.	Computation Time	Communication Overhead	Security Strength	Security Hardness	Security Requirements
Amin et al. [80]	0.801606301	0.636007828	0	0.666666667	0
Wang and Liu [69]	0	0	0	0	0
Li and Hong [70]	0.239640438	0	0	0	0
Jawaid et al. [83]	0.86112441	0.72407045	0	2.333333333	0
Mutaz et al. [71]	0	0.060665362	0.666666667	0	0.578947369
Lu et al. [72]	0	0	0.666666667	0	0.578947369
Li et al. [73]	0	0	0.666666667	0	0.578947369
Prameela & Ponmuthuramalingam [74]	0.523316496	0.060665362	0.666666667	0	0.578947369
Omala et al. [75]	0	0.060665362	0.666666667	0	0.578947369
Omala et al. [81]	0.761927561	0.636007828	0.666666667	0.666666667	0.578947369
Gao et al. [82]	0.722248821	0.459882583	0.666666667	0.666666667	0.578947369
Ullah et al. [84]	0.841285041	0.753424658	0	2.333333333	0
Jawaid et al. [76]	0	0.060665362	0.666666667	0	0.578947369
Noor et al. [85]	0.86112441	0.753424658	0	2.333333333	0
Hu et al. [77]	0	0	0.666666667	0	0.578947369

**Table 18:** Negative Distance from Average.

Authors & Ref. No.	Computation Time	Communication Overhead	Security Strength	Security Hardness	Security Requirements
Amin et al. [80]	0	0	1	0	1
Wang and Liu [69]	0.024824448	0.315068493	1	1	1
Li and Hong [70]	0	0.315068493	1	1	1
Jawaid et al. [83]	0	0	1	0	1
Mutaz et al. [71]	0.502226119	0	0	1	1
Lu et al. [72]	2.891748059	2.193737769	0	1	1
Li et al. [73]	0.59756282	0.315068493	0	1	1
Prameela & Ponmuthuramalingam [74]	0	0	0	1	1
Omala et al. [75]	0.537595855	0	0	1	1
Omala et al. [81]	0	0	0	0	1
Gao et al. [82]	0	0	0	0	1
Ullah et al. [84]	0	0	1	0	1
Jawaid et al. [76]	0.502226119	0	0	1	1
Noor et al. [85]	0	0	1	0	1
Hu et al. [77]	0.556088662	1.066536204	0	1	1

**Table 19:** Weighted Sum of PDA.

Authors & Ref. No.	Computation Time	Communication Overhead	Security Strength	Security Hardness	Security Requirements	$WP_{av}$
Amin et al. [80]	0.1603213	0.12720157	0	0.13333333	0	0.42085616
Wang and Liu [69]	0	0	0	0	0	0
Li and Hong [70]	0.0479281	0	0	0	0	0.04792809
Jawaid et al. [83]	0.1722249	0.14481409	0	0.46666667	0	0.78370564
Mutaz et al. [71]	0	0.01213307	0.133333	0	0.115789	0.26125588
Lu et al. [72]	0	0	0.133333	0	0.115789	0.24912281
Li et al. [73]	0	0	0.133333	0	0.115789	0.24912281
Prameela & Ponmuthuramalingam [74]	0.1046633	0.01213307	0.133333	0	0.115789	0.36591918
Omala et al. [75]	0	0.01213307	0.133333	0	0.115789	0.26125588
Omala et al. [81]	0.1523855	0.12720157	0.133333	0.13333333	0.115789	0.66204322
Gao et al. [82]	0.1444498	0.09197652	0.133333	0.13333333	0.115789	0.61888242
Ullah et al. [84]	0.168257	0.15068493	0	0.46666667	0	0.78560861
Jawaid et al. [76]	0	0.01213307	0.133333	0	0.115789	0.26125588
Noor et al. [85]	0.1722249	0.15068493	0	0.46666667	0	0.78957648
Hu et al. [77]	0	0	0.133333	0	0.115789	0.24912281

**Table 20:** Weighted Sum of NDA.

Authors & Ref. No.	Computation Time	Communication Overhead	Security Strength	Security Hardness	Security Requirements	$WN_{av}$
Amin et al. [80]	0	0	0.2	0	0.2	0.4
Wang and Liu [69]	0.0049649	0.0630137	0.2	0.2	0.2	0.66797859
Li and Hong [70]	0	0.0630137	0.2	0.2	0.2	0.6630137
Jawaid et al. [83]	0	0	0.2	0	0.2	0.4
Mutaz et al. [71]	0.1004452	0	0	0.2	0.2	0.50044522
Lu et al. [72]	0.5783496	0.43874755	0	0.2	0.2	1.41709717
Li et al. [73]	0.1195126	0.0630137	0	0.2	0.2	0.58252626
Prameela & Ponmuthuramalingam [74]	0	0	0	0.2	0.2	0.4
Omala et al. [75]	0.1075192	0	0	0.2	0.2	0.50751917
Omala et al. [81]	0	0	0	0	0.2	0.2
Gao et al. [82]	0	0	0	0	0.2	0.2
Ullah et al. [84]	0	0	0.2	0	0.2	0.4
Jawaid et al. [76]	0.1004452	0	0	0.2	0.2	0.50044522
Noor et al. [85]	0	0	0.2	0	0.2	0.4
Hu et al. [77]	0.1112177	0.21330724	0	0.2	0.2	0.72452497

**Table 21:** Final Ranking based on the chosen Parameters.

Authors & Ref. No.	$WP_{av}$	$WN_{av}$	$N\left(WP_{av}\right)$	$N(WN_{av})$	M	Rank
Amin et al. [80]	0.420856159	0.4	0.533015065	0.717732835	0.62537395	6
Wang and Liu [69]	0	0.667978588	0	0.528628944	0.264314472	14
Li and Hong [70]	0.047928088	0.663013699	0.060701007	0.532132507	0.296416757	13
Jawaid et al. [83]	0.783705639	0.4	0.992564569	0.717732835	0.855148702	3
Mutaz et al. [71]	0.26125588	0.500445224	0.330881031	0.646851863	0.488866447	9
Lu et al. [72]	0.249122807	1.417097166	0.315514473	1.866828710	0.157757237	15
Li et al. [73]	0.249122807	0.582526263	0.315514473	0.588929908	0.452222191	11
Prameela & Ponmuthuramalingam [74]	0.365919179	0.4	0.463437283	0.717732835	0.590585059	7
Omala et al. [75]	0.26125588	0.507519171	0.330881031	0.641860006	0.486370518	10
Omala et al. [81]	0.662043218	0.2	0.8384789	0.858866417	0.848672659	4
Gao et al. [82]	0.618882421	0.2	0.783815675	0.858866417	0.821341046	5
Ullah et al. [84]	0.785608606	0.4	0.994974681	0.717732835	0.856353758	2
Jawaid et al. [76]	0.26125588	0.500445224	0.330881031	0.646851863	0.488866447	8
Noor et al. [85]	0.78957648	0.4	1	0.717732835	0.858866418	1
Hu et al. [77]	0.249122807	0.724524973	0.315514473	0.488725974	0.402120224	12
